# Microscopy of the Teeth

**Published:** 1869-09

**Authors:** S. P. Cutler

**Affiliations:** Holly Springs, Miss.


### AMERICAN JOURNAL
OF
### DENTAL SCIENCE.

**Vol. III. THIRD SERIES--SEPTEMBER 1869. No. 5.**

### ARTICLE I.

### *Microscopy of the Teeth.*

By S. P. Cutler, D. D. S., M.D., Holly Springs, Miss.

**Read before the Southern Dental Association, July 30th, 1869.**

In this article I propose noticing a few points of an article in the May No. of the *American Journal of Dental Science* entitled "Dentine;" copied from the *Medical Investigator.*

The main points in the article accord with my views previously published, other points differ widely. It is to try and ferret out all the facts possible on the subject of the microscopic anatomy of the teeth that I take the liberty to review some points in the above article. While I take this liberty I freely allow others to do the same with me at any time, as I am after facts only. On Page 38 and 9 the writer states that the pulp cavity for a short distance from the foramina of the roots towards the crown, is lined or covered with osteal cells or cementum. Now if this be so the tubuli must first pass bodily through these osteal cells or layer of cementum, and then enter the dentine, then after passing through the dentine the nerve fibrils must enter the cement again, thereby traversing two strata of cement and one of dentine in their course from the pulp before reaching the surface of the fang. Still further up the fang he does not claim that the pulp cavity is covered by or lined by cement.

How can this be possible, for one portion of the pulp canal to be lined with cement and the balance not? Is this in accordance with nature? Is this in accordance with microsopic teachings? It certainly is not what my glass teaches, as I never have seen an instance of the kind from a large number of observations. Now in case the interior of the pulp cavity being lined with cement in accordance with all other cases the cement so lining must necessarily be covered by periosteum, or pericementum, the same as is similar to the exterior of the fang. Has this been found also to be the case? Undoubtedly it has not, if so, this would constitute Nature's botchwork and not her wonted harmony in all things which is so wonderful. I speak of the rule not of exceptions. I do not know what freaks she may be guilty of in many instances, I speak of the rule not the exceptions, though as above stated I have never seen an exception of this kind. I have in my possession two specimens, given, one at Cincinnati by Dr. Cushing of Chicago which he believed to be a lining of cement up a short distance from the foramen, but on careful examination under my instrument, I found nothing but true dentine though more irregular than usual in this instance. Again, how can it be possible that the minute opening at the apex, only large enough to admit of the passage of nerves and blood vessels, to be lined with periosteum? It certainly is impossible.

If such a case were possible there would have to be a new osteal basement membrane superadded on the inside of the pulp canal after dentification had ceased, thereby changing the whole plan of construction first laid down as the plan of operations for tooth development which we certainly have no very good grounds for believing, In laying the foundation or basement membrane, which is the true builder under all circumstances of all organic structures, for the development of the teeth, there commences, according to Beall, a double process of development, one travelling inward by depositing layer after layer of germinal matter, forming dentine on the one hand, and outwardly on the other forming enamel. Now passing downwards the same process may be recognized in neck and fang development, developing by the same basement membrane the dentine of the fang; but a different basement membrane to that of enamel is now laid down for the cement formation observing the same law of double development in the fang as in the crown, that is, commencing at the line of junction of cement and dentine where the enamel terminates one line of developmental march inwards the other outwards and downwards until the whole fang is fully developed and the tooth fully erupted. Up to this time the fang is open at apex and funnel shaped. By the time the tooth is fully developed the apex of fang begins to close up around the nerve pulp containing blood vessels, nerves and cellular tissue. By the time the apex is fully closed the dentine of the young tooth is fully completed for that period, the cement now having closed around the point of the fang down on to the pulp, as described above, but not following inwards, as, by this time, the door is closed and string pulled in, and no entrance here for foreign deposits of any kind. Up to this period there is but a thin layer of cement covering the fang uniformly, a few thicknesses throughout, but subsequently the cement gradually thickens at end of fang and on the fang for a third or more of its length with a regular taper, generally, no abrupt termination of additional layers of cells only in rare instances. We necessarily conclude that one basement membrane is not capable of building dentine, enamel and cement as they are all built upon separate and distinct types, and that a separate builder is needed for each. Hence a distinct basement membrane for each being in contact at the commencement of the osteal process, but continue to diverge or separate as the process of development advances and travels on *pari passu* until complete development. What other rational conclusion can we come to than that there is a separate and special basement membrane for each, two of which have no homologue in the system, that of the cement being common to other bones have homologous and non homologous basement membranes are represented in the development of teeth. As the cement thickens in advancing age and in some cases from other causes, the socket or alveolus necessarily gives way to the advancing encroachments of the hypertrophied fang. In this case it is but reasonable to suppose that where the fang is firm and fast in bone, that the resistance on the one hand is equal to the developing force of the cemental growth.

This, however, does not seem to be the case under all circumstances and we find the bone gradually yielding before the slow, sure tread of the progressing fang development. We may enquire how this was done; the rational answer must necessarily be by absorption, and here again we ask what is absorption of bone? Can we suppose that the bone on the one hand is removed and the same bone material so removed carried through the periosteum and deposited on the fang? No such thing, we must conclude that on the one hand bone is removed and carried out of the system, and, on the other, new deposits from food in the form of bone nutrition deposited as in another. or similar to other, bone development, only on a small scale and under different circumstances, though on a similar type and plan. The absorption of the alveolus I believe to be the work of an acid forming a soluble salt of lime which is washed away by the circulation and removed from the system mainly by the kidneys. This acid action no doubt being induced by the new condition of the fang, or it may depend on a removal of the inner walls of the socket from some obscure cause than by this acid action; and the fang development induced from necessity to prevent loosening and ultimate loss of tooth; otherwise though, the former hypothesis may be the most tenable. My conceptions relating to bone absorption being the joint action of oxygen or dentine and an acid on the lime, have already been published and need not be repeated in full in this article.

On Page 39. Says: "Some tubes give off no branches but run in an undulating manner without branching until they terminate at the base of the enamel." Here I shall again take issue, as no solitary tube having pulp cavity that does not branch one or more times just before entering the base of the enamel, forming what have been termed coronal branches. No properly prepared specimen but will show these branches distinctly under a good instrument, which fact also has been published by myself in a former article. Page 40, says: "There is an intertubular substance in dentine which is wholly composed of lime salts. These territories are without sensation. There are no fibrils there."

Says: "In dental decay we find portions of the cavity very sensitive to the touch of an instrument and other parts totally without sensibility." Does the writer here mean that the regions between the tubes are without sensibility and that the tubes themselves are all sensitive? If so, how is it possible to define these regions with an excavator when the tubes are probably not more on an average than the one five thousands of an inch apart, or even admit that they are the one thousand of an inch apart for sake of argument, how is it possible to trace out these distinctions along the meandering tubes with an excavator, say even the very smallest, which would more than cover on its cutting edge two or may be half a dozen of tubes and intertubular spaces at once. Such being the fact, I am unable to fully comprehend how any person can by any amount of training sharpen his sense of touch so as to make such nice discriminations, it certainly is a mystery to myself. I admit that some portions of cavity of a decayed tooth are frequently more sensitive than other portions in cases of exalted sensibility. These regions are just under the enamel, and in such the cement where the branches are most numerous, small and nearest the distal extremities or terminal points where sensation is most acute. This fact is not confined alone to teeth, but equally applies to all other sensitive regions of the body. The dentinal nerve fibrils through the whole course of the tubuli may be regarded as terminal fibrils capable of receiving throughout similar impressions or functions as each fibril stands separate, hence terminal throughout after leaving the pulp cavity.

Even in the pulp proper the nerve fibrils may be regarded terminal from the foramen, and they are all through the pulp more or less isolated though in close proximity in various regions of the pulp from plexuses filled and interspersed with cellular tissue. Not so in any other nerve cord where the fibrils are laid parallel and in close contact, and all inclosed in a neurilemma quite unlike the pulp membrane. The terminal points of fibrils are the true functional points and the nerve bundles, fasciculi or leashes constituting the nerve cord so termed, running from nerve centres to termini, are simple factors or carriers or conductors of force only similar to the conductive rods or wires of a battery tne terminal points only being those of greatest disturbance. No impressions are received or given off by the main trunk of any nerve ; not until the distal or points of filamental termination are reached.

The same may be said of arteries and veins, it is only in the capillary points where vascular physiology takes place or where the liquor sanguinis and oxygen, on the one hand leave the circulation as nutrition, and the products of waste on the other enter the vessels to be carried out of the organism.

These are the true vital points, and where both nerve fibrils and capillary blood vessels most generally meet with some few exceptions, as the dentinal nerve fibrils and corneal coat of the eye being familiar examples. Here also nutrition is eminently sustained. On page 40, the writer further remarks thus : The history of dentine shows that a tooth is not dead when pulp is dead, (*and removed from the tooth*) as we have seen that the dental tubes anastomose with the canaliculi of the cementum and the cementum cells are nourished from the periosteum of the roots with which they are united.

Falthus says : "Observation has taught us that a tooth may be *sensitive* after the removal of its nervous pulp the dentine within a cavity of decay proving extremely sensitive to the touch of an excavator in some instances, thus showing the presence of nerve fibrils received from the periosteum or dental periosteum.—*Medical Investigator.* Right here I take issue with the writer and deny the whole thing, and shall attempt to prove my position by the writers own *statement.* On the same page here is what he says: "There is an intertubular substance in dentine which is wholly composed of lime salts. These territories are *without sensation. There are no fibrils there.*"

Now let us analyze the conflicting statements He argues that the regions or spaces between the tubes have no nerve fibrils and no sensation. Now as there are no anastomosing of tubes, at least in the crown, or, in any that terminate under the enamel, how is it possible for sensibility to be transmitted from the dental periosteum when he admits the removal of the entire pulp? How is it possible for sensation to pass from the dental periosteum up through these regions of non-sensibility as no normal dentine will show any anastomosing with tubes ending under the enamel? How then is it possible when the nerve cavity is empty? It will be seen that the writer here contradicts himself. There must be some error somewhere in diagnosis. I am fully satisfied that when a nerve is killed or dies a natural death and has been removed bodily from the pulp cavity, the dentine at least of each tooth is *entirely* dead. The cement in such cases may retain sufficient vital connection with the dental periosteum to retain the tooth in its place for an indefinite period, without in many cases giving further trouble. I would recommend a careful repetition of the above cited cases on the part of the believers of this doctrine, then publish results.

We find regions in the most sensitive forms of decay where, even just under the enamel where a tooth is most generally sensitive, wholly devoid of sensibility, this can be readily accounted for from the fact that the more sensitive region have no live fibrils, their connection with the pulp having been removed by decay; where the microscopic anatomy of dentine is fully understood there need be no mystery connected with this fact. There is no true ganglia or gray matter found anywhere in the pulp of teeth, only fibrilla plexuses, heretofore described in a former article.

The dental branches, at least of the trigemina are both nutritive and sensorial the nearest ganglia to the teeth being Meckles and very small, the next and largest being within the cranium, the Casserian which furnishes general sensibility to the whole nerve, so that the dental are the most sensitive of the branches of this nerve.

Now let us take a retrospective view of pulp fibrils. beginning at the apex of fang. First.—Observe fibrils forming a central plexus through the pulp, these minor lateral ones which have heretofore been minutely described. Next observe those fibrils filled in and surrounded with soft cellular tissue throughout the pulp. Next observe fibrils passing through pulp membrane into cavity filled with fluid only *liquor sanguinis.* Next observe some fibrils passing this fluid into dentinal tubuli, ivory portion of tooth harder than bone. Now observe some fibrils passing through tubes in dentine through a nameless region, a *terra incognita* or *No Man's Land,* where the dentine and enamel unite by the remains of original basement membrane where ossification first commenced. Next observe fibrils terminating just into the base of enamel in a *cul de sac.* Next observe that after fibrils leave the pulp no anastomosing takes place anywhere in such as terminate under enamel and seldom in fang. Next observe fibrils not terminating under cement as in case of enamel, but passing bodily through to surface membrane. Now take into consideration the various media the dentinal nerve fibrils pass through and occupy, varying so widely in density and structures, and we find the anatomy and physiology of the teeth to be perhaps the most wonderful of all structures, when we consider the almost exclusive use of these organs to be mechanical and laborious, simply a set of grinders and cutters or choppers, in short "hewers of wood and drawers of water," which may be replaced by artificial substitutes quite successfully.

